# Validation of a diabetes risk score in identifying patients at risk of progression to abnormal glucose tolerance post partum

**DOI:** 10.1186/1753-6561-6-S4-O36

**Published:** 2012-07-09

**Authors:** C Crowe, E Noctor, LA Carmody, B Wickham, G Avalos, G Gaffney, P O’Shea, F Dunne

**Affiliations:** 1 Department of Medicine, University Hospital Galway and National University of Ireland, Galway, Ireland; 2 Department of Obstetrics and Gynaecology, University Hospital Galway, Ireland; 3 Department of Clinical Biochemistry, University Hospital Galway, Ireland

## Introduction

FINDRISC (Finnish Diabetes Risk Score) is a risk assessment tool widely used for the prediction of the development of type 2 diabetes (T2DM), combining a questionnaire with simple anthropometric measurements to identify patients at risk of developing diabetes, with increasing score (0-26) signifying increased risk. A cut off score of 9 has previously been proposed (with drug-treated DM as the endpoint) with a positive predictive value (PPV) of 0.12 and negative predictive value (NPV) of 0.99, area under receiver operating characteristic curve (AuROC)=0.80. It has been well validated in the general population.

## Methods

We examined its use in predicting progression to pre-diabetes/diabetes in a cohort of Caucasian patients with a history of gestational diabetes mellitus (GDM). 116 women with a history of GDM underwent screening 1-5 years post-index pregnancy. Those with a history of persistent post-partum dysglycaemia had fasting glucose levels taken, while others underwent a 75g OGTT.

## Results

Of the 116 women with a history of GDM, 83 showed normal glucose tolerance (NGT) post-partum (71.6%). 22 patients had abnormal OGTT at 12 weeks (18.9%). A further 11 patients (9.5%) had pre-diabetes/diabetes at rescreening. The FINDRISC score was higher in patients with pre-diabetes/diabetes than those with NGT post partum (mean score 13.6+/- 4.1 vs. 11.0+/- 3.7, p<0.01). For a cut-off score of 9, PPV was 0.30, NPV was 0.79, AuROC=0.69, comparable with published data in the general population.

## Conclusions

This study shows the validity of an inexpensive, convenient risk score in helping to determine which patients may need more frequent screening post GDM.

